# Environmental, Occupational, and Demographic Risk Factors for Clinical Scrub Typhus, Bhutan

**DOI:** 10.3201/eid2905.221430

**Published:** 2023-05

**Authors:** Tandin Zangpo, Yoenten Phuentshok, Kezang Dorji, Chencho Dorjee, Sithar Dorjee, Peter Jolly, Roger Morris, Nelly Marquetoux, Joanna McKenzie

**Affiliations:** Khesar Gyalpo University of Medical Sciences of Bhutan, Thimphu, Bhutan (T. Zangpo, Y. Phuentshok, K. Dorji, C. Dorjee, S. Dorjee);; Massey University, Palmerston North, New Zealand (T. Zangpo, Y. Phuentshok, K. Dorji, P. Jolly, N. Marquetoux, J. McKenzie);; Ministry of Health, Thimphu (T. Zangpo, K. Dorji);; World Organisation for Animal Health, Paris, France (Y. Phuentshok);; Morvet Ltd, Masterton, New Zealand (R. Morris)

**Keywords:** scrub typhus, environmental risk factor, occupational risk factor, demographic risk factor, clinical scrub typhus, Orientia tsutsugamushi, bacteria, vector-borne infections, risk factors, epidemiology, case‒control, One Health, cardamom, zoonoses, Bhutan

## Abstract

Underdiagnosis and underreporting of scrub typhus has increasingly affected public health in Bhutan since its initial detection in 2008. Identifying scrub typhus risk factors would support early diagnosis and treatment for this nonspecific febrile disease, reducing the incidence of potentially fatal complications. We conducted a hospital-based, case‒control study during October‒December 2015 in 11 scrub typhus‒prone districts. We identified harvesting cardamom as the major risk factor (odds ratio 1,519; p<0.001); other factors were traditional housing, largely caused by an outside toilet location, as well as owning a goat and frequently sitting on grass. Harvesting vegetables, herding cattle in the forest, and female sex were protective. Age had a nonlinear effect; children and the elderly were more likely to seek treatment for clinical scrub typhus. This study has informed public health policies and awareness programs for healthcare workers through development of National Guidelines for Prevention, Treatment and Control of Scrub Typhus in Bhutan.

Scrub typhus, or tsutsugamushi disease, is a vectorborne zoonotic disease caused by *Orientia tsutsugamushi*, which is endemic to the tsutsugamushi triangle, centered in Southeast and Pacific Asia (*1*). Trombiculid mites of the genus *Leptotrombidium* are reservoir and vector for *O. tsutsugamushi* (*2*). The infective larvae, called chiggers, transmit the pathogen to humans as incidental hosts. Rodents are maintenance hosts for mites; high numbers increase mite abundance and risk for scrub typhus where the pathogen is present (*2*).

The resurgence and reemergence of scrub typhus in disease-endemic areas (*3*,*4*) places >1 billion persons at risk for this disease globally; an estimated 1 million new cases of scrub typhus occur each year (*4*–*6*). Scrub typhus manifests as a nonspecific febrile illness that is difficult to diagnose. Early treatment using doxycycline or chloramphenicol usually results in rapid remission, but delays in diagnosis and treatment are associated with potentially fatal complications (*7*–*9*). A median case-fatality rate of 6% in untreated patients has been reported, and death rates can be up to 70% (*4*).

In Bhutan, scrub typhus was first detected in 2008 among a cluster of pyrexia of unknown origin cases reporting to Gedu Hospital in Chukha District (*10*). The disease became reportable in 2010, and annual reports have regularly increased, particularly those from southern subtropical regions. A descriptive study showed that scrub typhus is a major cause of febrile illness in Bhutan, albeit underdiagnosed and underreported, presumably because of lack of healthcare worker awareness of the disease and differential diagnostic challenges (*11*). Scrub typhus was most commonly diagnosed in rural, elderly persons and students in southern districts of Bhutan; the highest incidence was in summer and autumn, during July‒November (*11*). The high-incidence period aligns with the monsoon season, presumably associated with a higher density of chiggers (*2*) and a higher risk for exposure through intensive agricultural activities. Further epidemiologic studies are required to provide robust evidence of risk factors for scrub typhus in Bhutan, which would support public health action to prevent and control the disease.

The objective of our study was to identify risk factors for clinical scrub typhus in patients seeking treatment at healthcare centers in scrub typhus‒endemic areas of Bhutan. Results from this study were intended to inform public health policies and contribute to raising awareness about scrub typhus among clinicians and populations at risk.

## Materials and Methods

**Figure 1:**
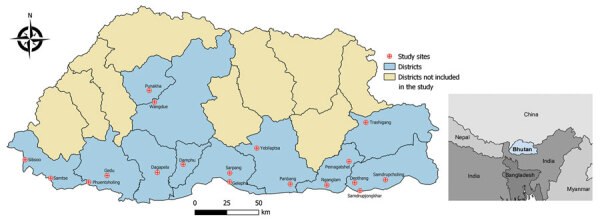
Location of 18 healthcare centers in 11 districts that had high reporting rates for scrub typhus that were included in the case‒control study conducted in Bhutan, 2015.

We conducted a hospital-based, case‒control study during October‒December 2015, coinciding with autumn and the later months of the high-risk period for scrub typhus in Bhutan. The study was conducted in 11 of 20 districts in Bhutan that had reported the highest numbers of scrub typhus cases in the 5 years after scrub typhus was made a reportable disease in 2010. Study districts were located in the southern and central regions, at altitudes ranging from 173 m to 1,576 m above sea level. All 18 healthcare centers in those districts participated in the study, including 15 hospitals (secondary and tertiary healthcare centers) and 3 basic health units (primary healthcare centers) (Figure 1).

We recruited case-patients and controls from patients >1 year of age who came to the outpatient departments of the 18 healthcare centers. For each case-patient, we enrolled 3 controls from the same healthcare center, 2 matched by village and 1 unmatched. Matching controlled for the confounding effect of village-level risk factors, enabling investigation of occupational, recreational and household-related exposures of persons. Unmatched controls enabled investigation of individual exposures and spatially correlated variables such as urban/rural and possibly occupation (*12*).

Provisional case-patient definition was a patient consulting the outpatient department during the study period who met 3 criteria. Those criteria were febrile illness (axillary temperature >37.5°C) with >1 of the following signs/symptoms: eschar, rashes, headache, cough, general malaise, myalgia, or lymphadenopathy; having a positive test result on the ST Rapid Diagnostic Tsutsugamushi Test (RDT; Bioline, https://www.bioline.com) at the hospital on the visit day; and residing in the study district for the previous month or longer. Confirmed case-patient definition was a provisional patient who had a positive test result for a subsequent serum ELISA (Scrub Typhus Detect IgM ELISA System; InBios, https://inbios.com) conducted at the Royal Center for Disease Control in Thimphu, Bhutan.

Provisional controls were enrolled from patients who visited the same outpatient department on the same day or in the next few days (maximum 1 week) after recruitment of the provisional case and who met 2 criteria: no history of febrile illness or any of the signs/symptoms described for case eligibility in the previous month and a negative test result for the scrub typhus RDT on the visit day. Two provisional matched controls were selected for each provisional case-patient on the basis of their residing in the same village. One provisional unmatched control was selected for each provisional case-patient from patients visiting the same outpatient department who resided anywhere in the study districts, excluding the case-patient’s village. Confirmed controls were provisional controls who had a negative result for subsequent serum ELISA.

Excluding provisional case-patients after a negative result for the ELISA resulted in excluding their provisional unmatched and matched controls. In a similar fashion, after excluding provisional controls based on a positive ELISA result, any case-patients who no longer had >1 matched control were also excluded.

The sample size was estimated at the design stage based on the magnitude of association of known risk factors from disease-endemic regions reported in the literature. A similar case‒control study in China identified various exposures to crops, grasslands, and outdoors activities as risk factors for scrub typhus (*13*). The average odds ratio (OR) of all significant risk factors in that study was 2.5. For our study, assuming a 10% prevalence of exposure for the target population and aiming to detect an OR >2.5 with 95% confidence and 80% power, we required a sample size of 92 case-patients and 276 controls. We increased this number by 10% to include nonresponse or missing information and aimed to enroll >100 case-patients and 300 controls.

Blood samples from provisional case-patients and controls were obtained by trained laboratory technicians. The RDT was performed on serum samples at the healthcare center on the same day as sample collection. The SD Bioline Tsutsugamushi Test is a commercial point-of-care rapid diagnostic test that detects IgG, IgM, and IgA against *O. tsutsugamushi* (*14*) and is used in all hospitals in Bhutan to support clinicians’ differential diagnosis and timely treatment for scrub typhus. Aliquots of serum samples were frozen at −4°C to −8°C and transported to the Royal Center for Disease Control for ELISA testing (InBios Scrub Typhus Detect IgM ELISA System). For the ELISA, we used an optical density (OD) >0.54 as the cutoff for positivity, based on regional cutoff values for OD readings in scrub typhus studies conducted in Thailand and India (*15*–*17*).

We trained healthcare workers from participating healthcare centers to enroll study participants and administer questionnaires. We used a structured, standardized, pretested questionnaire with closed questions in English to interview provisional case-patients and controls at the time of recruitment. We collected information on demographic characteristics, house details, animal ownership, environmental and occupational exposures. Given a scrub typhus incubation period of 21 days, questions were related to a potential exposure period of 1 month before recruitment.

We conducted descriptive analysis of demographic variables of confirmed case-patients and controls. We analyzed data for case-patients and matched controls by using conditional logistic regression and data for case-patients and unmatched controls by using standard logistic regression. We omitted variables with >10% missing information, as well as categorical variables with responses to only 1 level. We conducted initial univariable analysis for each dataset. We considered variables that had a χ^2^ p value for the likelihood ratio test (variance comparison with the null model) <0.2 in univariable analyses for multivariable analyses. Variables that contained too few responses were recategorized. For multivariable analysis, we used backward and forward variable selection and compared models by using the likelihood-ratio test (χ^2^ test, 5% significance level).

We explored collinearity between related variables. For correlated variables, we selected the variable with the most significant contribution to the model based on the likelihood ratio test to avoid multicollinearity in the final model. We tested 2-way interactions between significant variables. We tested linearity of continuous variables (e.g., age) by using polynomials and retained the best fitting model for each analysis to estimate adjusted ORs for scrub typhus risk factors. We performed data management and analyses in R software (The R Project for Statistical Computing, https://www.r-project.org).

Inclusion in the study was based upon patients’ written consent. We coded samples to protect patient identity. There was no interruption of laboratory and treatment services because of this study. Our study was approved by the Research Ethics Board of Health, Ministry of Health, Bhutan.

## Results

**Figure 2:**
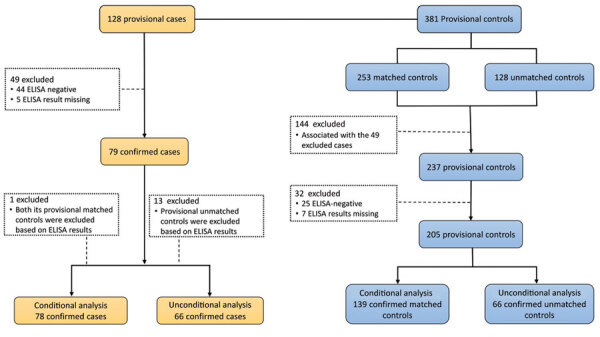
Selection process for provisional cases and controls for conditional and unconditional analyses based on confirmatory ELISA results, Bhutan, 2015.

We recruited 128 provisional scrub typhus case-patients and 381 provisional controls at the 18 healthcare centers during the 3-month study period. After confirmatory ELISA testing, we retained 78 confirmed case-patients and 139 confirmed matched controls for conditional analysis and 66 confirmed case-patients and unmatched controls for unconditional analysis (Figure 2). Of the 128 RDT-positive provisional case-patients, ELISA results were available for 123, of which 79 (64%) were confirmed ELISA-positive (OD >0.54) case-patients. Of the 237 RDT-negative provisional controls who were associated with case-patients retained for the analysis, ELISA results were available for 230, of which 205 (89%) were confirmed ELISA-negative (OD<0.54) controls.

**Figure 3:**
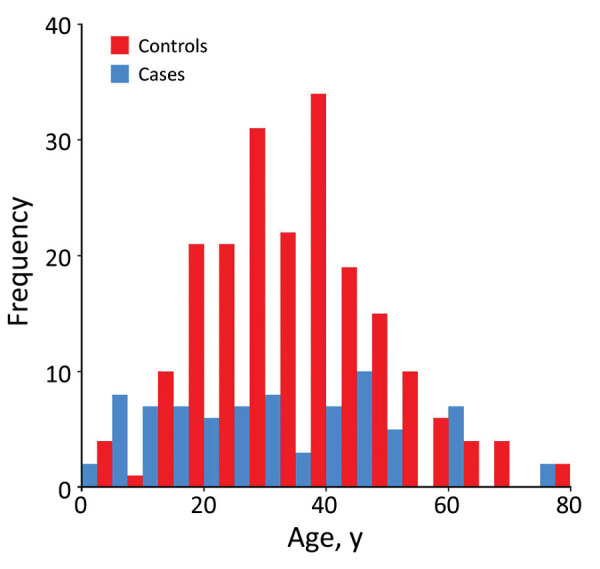
Age distribution of confirmed cases and controls included in a study of risk factors for clinical scrub typhus in disease-endemic areas of Bhutan, 2015.

Most (52%) study participants were farmers, equally represented in case-patients and controls (Table 1). The next most common categories were children and housewives, with a higher proportion of children as case-patients and a higher proportion of housewives as controls. There were more female (59%) than male participants. The median age was 28 years for case-patients and 35 years for controls. The age distribution for case-patients and controls was different; the middle-age category was overrepresented among controls (Figure 3).

**Table 1:** Distribution of occupation and sex for confirmed case-patients and controls in a study of risk factors for clinical scrub typhus in disease-endemic areas, Bhutan, 2015

Variable	No. (%) controls	No. (%) case-patients	Total
Occupation			
Farmer	108 (53)	39 (50)	147
Housewife	34 (17)	4 (5)	38
Preschool/student	20 (10)	23 (29)	43
Civil servant/military	19 (9)	2 (3)	21
Corporate/private sector	12 (6)	4 (5)	16
Construction worker	9 (4)	2 (3)	11
Monk/nun	3 (1)	4 (5)	7
Sex			
F	126 (61)	40 (51)	166
M	79 (39)	38 (49)	117

**Figure 4:**
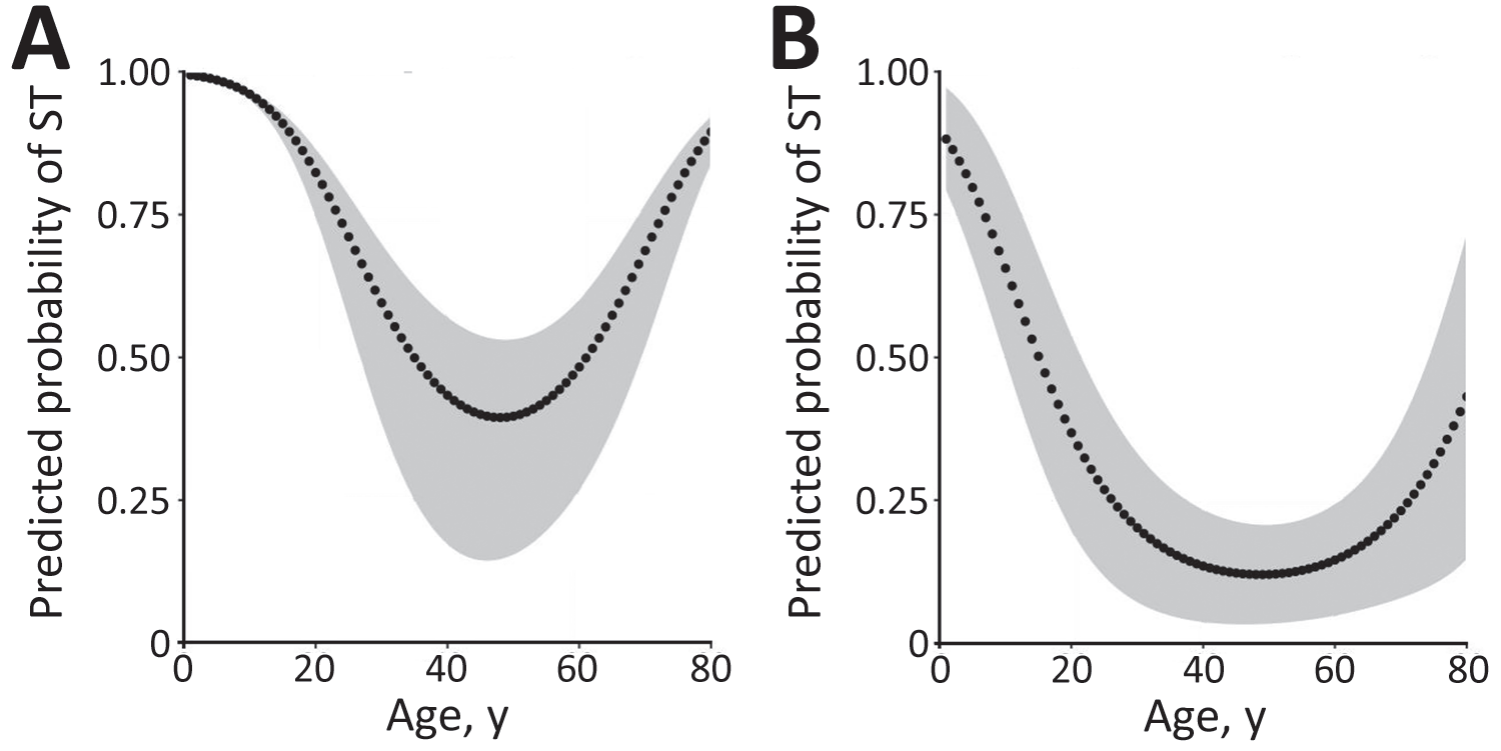
Effect of age on the probability of presenting to hospitals with a clinical case of ST in a group of matched cases and controls (A) and a second group of unmatched cases and controls (B), Bhutan, 2015. Gray areas indicate 95% CI. ST, scrub typhus.

We compiled results for conditional (Table 2) and unconditional (Table 3) univariable analyses and significant variables in the best-fitting conditional (Table 4) and unconditional (Table 5) multivariable models. Harvesting cardamom in the previous month was the major risk factor for clinical scrub typhus: OR 1,519 (95% CI 26.23–8,805.98) in conditional analysis (Table 4) and a smaller OR of 5.98 (95% CI 0.86–41.46) in unconditional analysis (Table 5). Traditional housing had a high OR of 472.3 (95% CI 17.28–12,900) in conditional analysis, and an additional independent risk was associated with having no shower in the house. A closely related variable, toilet outside the house, had a significant OR of 10.65 (95% CI 2.37–47.84) in unconditional analysis. Sitting or sleeping on the grass >10 times in the previous month had a high OR in both analyses. We found a significant, independent quadratic effect of age in both models, with a higher predicted probability of clinical scrub typhus for children and older persons after controlling for the effect of other variables (Figure 4).

**Table 2:** Variables that had a χ^2^ p value <0.2 in conditional univariable analysis that were considered for the final multivariable model in a study of risk factors for clinical scrub typhus, Bhutan, 2015

Variable*	Odds ratio	Wald p value	χ^2^ p value	Multivariable model
Demographic variables				
Age (continuous)	0.98	0.020	0.016	†
Female sex (reference: male)	0.59	0.059	0.057	
Occupation (reference: farmer)			<0.001	
Housewife	0.32	0.086		
Civil servant/military	0.31	0.209		
Corporate/private business	0.86	0.848		
Preschool/student	4.79	0.002		
Monk/nun	6.09	0.145		
Construction worker	0.26	0.235		
Rural residence (reference: urban)	4.00	0.092	0.063	
House-related variables				
Traditional house (reference: modern)	3.03	0.006	0.004	†
Shower located outside (reference: inside)	2.35	0.007	0.007	†
Toilet located outside (reference: inside)	5.21	0.032	0.011	
Flooring of mud/clay (reference: no)	2.08	0.079	0.075	
Water supply from a stream (reference: no)	4.59	0.070	0.053	
Water supply community pipe (reference: no)	0.54	0.150	0.149	
Water supply private pipe (reference: no)	1.77	0.162	0.159	
Saw rodents in house (reference: no)	3.02	0.017	0.009	
Animal ownership				
Possessed a cat (reference: no)	1.53	0.171	0.168	
Possessed poultry (reference: no)	1.78	0.081	0.077	
Possessed a goat (reference: no)	2.77	0.023	0.019	†
Protective measures				
Wore footwear outdoors (reference: never)			0.060	†
Sometimes (occasionally and most of the time)	5.55	0.047		
Always	3.17	0.159		
Used insect repellent on clothes occasionally (reference: never)	0.22	0.179	0.128	
Bitten by ticks or fleas (reference: no)	1.57	0.165	0.163	
Cultivation-related exposures				
Harvested cardamom (reference: no)	4.15	0.007	0.004	†
Harvested vegetables (reference: no)	0.27	0.004	0.002	†
Worked in vegetable garden (reference: no)	0.42	0.009	0.006	
Worked in mixed crop fields (reference: no)	0.26	0.096	0.066	
Worked in rice paddy (reference: no)	1.79	0.194	0.189	
Worked in an orchard (reference: no)	2.15	0.124	0.120	
Forest-related exposures				
Household children <12 y of age played in the bush (reference: never)			0.045	
Occasionally	8.04	0.214		
Daily	4.07	0.302		
Did not have children <12 y of age	1.28	0.842		
Collected forest products (reference: no)	2.39	0.086	0.086	
Visited forest for any reason (reference: no)	1.71	0.096	0.089	
Undertook reafforestation work (reference: no)	2.64	0.122	0.110	
Cleared scrub for others as a job (reference: no)	1.63	0.125	0.123	
Herded cattle in the forest (reference: no)	0.47	0.080	0.068	†
Other outdoor exposures				
Sat or slept on grass (reference: never)			0.053	†
1–5 times	1.69	0.185		
6–10 times	2.74	0.066		
>10 times	3.29	0.011		

*Exposure variables related to a period of 1 mo before patients’ recruitment at healthcare center. †Significant (p<0.05) in the multivariable model.

**Table 3:** Variables that had a χ^2^ p value <0.2 in unconditional univariable analysis that were considered for the final multivariable model in a study of risk factors for clinical scrub typhus, Bhutan, 2015

Variable*	Odds ratio	Wald p value	χ^2^ p value	Multivariable model
Demographic variables				
Age (continuous)	0.98	0.032	0.028	†
Occupation (reference: farmer)			0.036	
Housewife	0.31	0.066		
Civil servant/military	0.19	0.043		
Corporate/private business	0.64	0.582		
Preschool/student	1.48	0.387		
Other (monk/nun, construction)	2.57	0.424		
Rural location (reference: urban)	7.23	<0.001	<0.001	
House-related variables				
Traditional house (reference: modern)	3.75	0.001	<0.001	
2‒3 floors in house (reference: 1 floor)	0.40	0.017	0.015	
Traditional toilet‡ (reference: modern)	2.18	0.046	0.043	†
Toilet located outside house§ (reference: inside)	11.23	<0.001	<0.001	
Toilet type (reference: modern inside)			<0.001	
Modern outside	11.57	0.002		
Traditional outside	16.20	<0.001		
Kept piles of wood (firewood, logs/timber, brush, and twigs) against the house wall (reference: no)	1.88	0.078	0.076	
Kept firewood against house wall (reference: no)	1.85	0.100	0.098	†
Kept logs/timber against house wall (reference: no)	2.07	0.151	0.143	
Kept wood in the yard (reference: no)	0.48	0.047	0.045	
Animal ownership				
Possessed cattle (reference: no)	0.44	0.023	0.021	
Possessed poultry (reference: no)	0.48	0.037	0.035	
Protective measures				
Worked outdoors in short sleeves (reference: never)			0.035	
Occasionally	0.94	0.912		
Most of the time	0.59	0.299		
Always	2.71	0.091		
Worked in garden/fields with bare hands (reference: never)			0.108	†
Occasionally	3.26	0.040		
Most of the time	1.47	0.575		
Always	0.95	0.911		
Changed working clothes to sleep (reference: never)			0.119	
Occasionally	0.62	0.536		
Most of the time	0.25	0.080		
Always	0.60	0.499		
Frequency of showers (reference: never and weekly)			0.120	
2–3 times/week	0.47	0.069		
Every day	0.48	0.104		
Cultivation-related exposures				
Harvested cardamom (reference: no)	7.11	0.013	0.003	†
Worked in an orchard (reference: no)	1.86	0.180	0.174	
Cleared bushes for others as a job (reference: no)	2.13	0.056	0.053	†
Forest-related variables				
Visited forest (Reference: no)	2.26	0.023	0.022	
Collected timber in the forest (reference: no)	4.19	0.205	0.157	
Collected nonwood products in forest (reference: no)	2.90	0.129	0.109	
Other outdoor exposures				
Sat or slept on grass (reference: never)			0.059	†
1– 6 times	1.43	0.436		
7–10 times	1.67	0.392		
>10 times	3.47	0.009		

*Exposure variables related to a period of 1 mo before patients’ recruitment at healthcare center. †Significant (p<0.05) in the multivariable model. ‡Traditional toilets are a pit latrine with wattle and daub walls or planks without continuous water supply. Modern toilets have an Indian or western pot type toilet with concrete or tiled walls and floor with a continuous water supply. Toilet type was considered regardless of location inside or outside the house. §Considered location only, regardless of modern or traditional toilet type.

**Table 4:** Significant variables in best-fitting model for conditional multivariable analysis of laboratory-confirmed clinical case-patients who had scrub typhus and unmatched controls, Bhutan, 2015*

Variable†	OR (95% CI)	p value
Age (order 1 term)	NA	0.001
Age (quadratic age)	NA	0.007
Female (reference: male)	0.19 (0.05‒0.67)	0.010
Traditional house type‡ (reference: modern)	472.3 (17.28–12,900)	<0.001
Shower located outside house (reference: inside)	6.21 (1.28–30)	0.023
Sat or slept on grass (reference: never)		
1–10 times	1.81 (0.51–6.5)	0.360
>10 times	16.38 (1.68–160.05)	0.016
Wore footwear outdoors (reference: never)		
Sometimes	9.25 (1.09–78.37)	0.041
Always	0.88 (0.12–6.31)	0.898
Herded cattle in the forest (reference: no)	0.06 (0.01–0.52)	0.010
Possessed a goat (reference: no)	36.52 (3.59–371.91)	0.002
Harvested vegetables (reference: no)	0.03 (0.00–0.28)	0.002
Harvested cardamom (reference: no)	1,519.00 (26.23–88,005.98)	<0.001

*NA, not applicable; OR, odds ratio. †Exposure variables related to a period of 1 mo before patients’ recruitment at healthcare center. ‡Traditional Bhutanese houses are commonly 2-storied structures made from wood and earthen material, namely wattle and daub interior walls, with stone and earth retaining walls and wooden plank roofing. Modern houses are generally multistoried structures made of concrete materials, such as bricks, cement, and iron rods with minimal use of wooden frames for windows and flooring with zinc sheet roofing.

**Table 5:** Significant variables in best-fitting model for unconditional multivariable analysis of laboratory-confirmed clinical case-patients who had scrub typhus and unmatched controls, Bhutan, 2015*

Variable†	OR (95% CI)	p value
Age (order 1 term)	NA	0.001
Age (quadratic term)	NA	0.023
Toilet outside the house (reference: toilet inside)	10.65 (2.37‒47.84)	0.002
Kept logs of wood against the wall (reference: no)	4.17 (1.18‒14.71)	0.027
Sat or slept on grass (reference: never)		
1–10 times	2.08 (0.69‒6.31)	0.195
>10 times	6.06 (1.56‒23.59)	0.009
Cleared bushes for others as a job (reference: no)	4.71 (1.61‒13.82)	0.005
Worked outdoors (garden/fields) with bare hands (reference: never)		
Sometimes	4.18 (1.03‒17.01)	0.045
Always	0.87 (0.26‒2.90)	0.824
Harvested cardamom (reference: no)	5.98 (0.86‒41.46)	0.070

*NA, not applicable; OR, odds ratio. †Exposure variables related to a period of 1 mo before patients’ recruitment at healthcare center.

Conditional analysis showed that possessing a goat was a major risk factor for scrub typhus (OR 36.52, 95% CI 3.59–371.91) but that herding cattle in the forest was strongly protective (OR 0.06, 95% CI 0.01–0.52). Being female and harvesting vegetables were also protective. Unconditional analysis showed that storing wood logs against the house and clearing bush had a higher risk for scrub typhus. Persons who sometimes worked outdoors with bare hands also had a higher risk compared with persons who never worked outdoors with bare hands.

## Discussion

This study identified major occupational, recreational, household-related, animal ownership, and demographic risk factors for scrub typhus among ill patients who sought treatment during the latter months of the high-incidence period in Bhutan during 2015. Clinical diagnosis of scrub typus is challenging because of its nonspecific clinical manifestations. Furthermore, previous infections, cross-reactivity, and low accuracy of routine diagnostic tests are likely to complicate the diagnosis of scrub typhus (*18*). Thus, identifying risk factors for this disease is a valuable aid for accurate clinical diagnosis and instigation of early treatment, reducing the risk for potentially fatal complications.

Harvesting cardamom was the highest risk factor for scrub typhus, which had a conditional OR of 1,519.00 (95% CI 26.23–88,005.98) (Table 4). Cardamom is a short, bushy plant that might provide favorable mite habitat. It is grown throughout the southern districts of Bhutan, preferring shaded conditions on hilly terrain with moist soils (*19*). Cardamom harvest occurs during August‒September in low and middle altitudes and November‒December in high altitudes, coinciding with the scrub typhus risk period (*11*) and with our study period (October‒December). Hilly terrain and autumn harvest also appeared as major risk factors for scrub typhus in China (*13*) and Japan (*20*). Growing cardamom is a lucrative business; its cultivation has been increasing in Bhutan since 2013 (*19*), which is likely to have contributed to the increasing incidence of scrub typhus. Cardamom is also cultivated in nearby countries (India [*21*] and Nepal [*22*]), where it might also contribute to increased incidence of scrub typhus (*23*,*24*).

Harvesting vegetables was negatively associated with scrub typhus in our study, contrasting with findings of a study in China conducted during the same time of year in which working in vegetable fields increased the risk for scrub typhus (*13*). A possible explanation is that vegetable species or growing environment differed between the 2 countries. In our study, vegetables harvested included broccoli, tomatoes, and onions, which might have been grown in an environment with minimal mite habitat, thus reducing the risk for scrub typhus.

Traditional housing was another strong risk factor in conditional analysis (OR 472.30, 95% CI 17.28–12,900.00). This variable was correlated with having an outside toilet; however, it captured more of the variability in scrub typhus risk than toilet location and was retained in the final model. In contrast, in unconditional analysis, having an outside toilet explained more variability in scrub typhus risk than traditional house type. Having an outside toilet, regardless of whether the toilet was modern or traditional type, might directly increase exposure to mites from surrounding bushes, or it might be a proxy for other factors associated with traditional housing. Having no shower in the house was a major risk factor in conditional analysis, independent of house type. This result might be related to a finding from a study in India that bathing after work and changing clothes before sleep were protective measures (*25*). Traditional housing also might be more prone to rodent infestation than modern housing, which might increase the abundance of infected mites in the living area (*2*). Keeping wood logs against the house was a major risk factor in unconditional analysis, consistent with findings from a study in India (*25*) and possibly reflecting exposure while collecting wood or representing a suitable environment for rodents. Related findings from other studies include house yards without cement flooring (*13*) and poor sanitary conditions (*26*).

Frequently sitting or sleeping on grass was strongly associated with scrub typhus in both analyses, consistent with findings of previous studies (*2*). Owning a goat was a major risk factor identified in conditional analysis, but herding cattle in the forest was associated with a reduced risk. Other studies have found evidence of both scrub typhus seropositivity (*27*) and chigger infestation of goats (*28*). Thus, the association with owning a goat might be related to exposure to infected mites carried by goats or in the goats’ feeding environment. Given that goats are browsers and cattle are grazers, goat herders might have been more exposed to low-lying bushes, the preferred habitat of trombiculid mites, whereas cattle herders might have been more exposed to grassy patches in the forest that were clear of bushes, thus reducing their risk for scrub typhus.

Although wearing gumboots at work was associated with a reduced risk for scrub typhus in India (*25*), conditional analysis in our study found that sometimes wearing footwear outdoors was associated with a higher risk for scrub typhus than never wearing footwear outdoors. This paradoxical outcome might have been influenced by formulation of the question (i.e., the reference category included persons who never worked outdoors, as well as persons who never wore footwear outdoors). Whereas the study from India (*25*) found no protective effect of wearing gloves while working outdoors, unconditional analysis in our study found that sometimes working outdoors with bare hands was associated with a higher risk than never working outdoors with bare hands. However, this result might not reflect a real protective effect of covering hands because it might also have been influenced by the reference category including persons who never worked outdoors and persons who never worked outdoors with bare hands.

In both analyses, we found a significant nonlinear effect of age; the risk for scrub typhus was higher for young children and the elderly, suggesting an increased exposure to infected mites in these age groups through age-related behaviors, or, alternatively, that the young or elderly are more susceptible to developing clinical scrub typhus. A study in India highlighted the usefulness of including scrub typhus in the differential diagnosis of pyrexia of unknown origin in children unresponsive to treatment with common antimicrobial drugs (*9*). The conditional analysis also indicated that female study participants had a lower risk for scrub typhus, consistent with a sex effect observed in China (*13*). This finding might reflect less frequent involvement in outdoor activities compared with men and boys, reducing risk for exposure to mites.

The findings of this study are relevant for patients who have clinical scrub typhus and visited a healthcare center during the later months (October‒December) of the risk period for this disease. Thus, these persons might underrepresent the strength of risk or the full extent of risk factors associated with *O. tsutsugamushi* infection throughout the July‒November risk period. Also, our study did not include infected persons who did not visit a healthcare center (*2*).

The RDT used to recruit case-patients who had scrub typhus has been reported to have a low sensitivity of 38% (95% CI 28%–49%) (*14*). This test detects IgG, IgM, and IgA against *O. tsutsugamushi* , so it might detect historical as well as active scrub typhus cases (*14*), resulting in persons who have historical infections to be included as provisional case-patients. The in-series ELISA targeting IgM would have increased the classification accuracy of the case-patient and control groups, given the high sensitivity (99.9%, 95% CI 90.4–100.0) and specificity (99.1%, 95% CI 96.8%–99.8%) reported for that test (*14*). Any remaining misclassification in case-patient or control groups would contribute to biasing the results toward the null, but that did not preclude the study from identifying major risk factors.

In this study, we compared case-patients with a group of controls matched by village and a group of unmatched controls. Matching increased the probability that case-patients and controls had the same potential exposure to infected chiggers in the vicinity of their residential environment, enabling investigation of individual and household-related risk factors. The unmatched controls enabled an investigation of variables that might be spatially correlated, such as rural versus urban location and possibly occupation. Although conditional and unconditional analyses identified age, traditional house type or toilet location, harvesting cardamom, and sitting on the grass as risk factors, conditional analysis produced much larger ORs for most variables and identified additional risk factors: owning a goat, being female, harvesting vegetables, and herding cattle in the forest. Distribution and density of mites is known to vary throughout an area (*2*), which might have affected the power of the unmatched analysis. Case-patients and unmatched controls might not have had the same exposure to infected mites; thus, unmatched controls might have had similar recreational and occupational factors as case-patients but in environments where there were no infected mites.

This study has contributed major public health benefits by providing strong evidence for occupational, environmental, and demographic risk factors that can support early diagnosis and treatment for scrub typhus, reducing the incidence of complications and deaths. Many health workers were not aware of scrub typhus, and study findings contributed to public health policy and awareness raising among clinicians, particularly in areas with a high disk for this disease.

Recommendations from this study were made to the Department of Public Health and contributed to new National Guidelines for Prevention, Treatment and Control of Scrub Typhus in Bhutan. The findings of this study could also be useful for improving awareness, and early diagnosis and treatment in nearby countries in regions where scrub typhus is also an emerging disease of concern.

## References

[1] Yang HH, Huang IT, Lin CH, Chen TY, Chen LK. New genotypes of *Orientia tsutsugamushi* isolated from humans in Eastern Taiwan. PLoS One. 2012;7:e46997. 10.1371/journal.pone.0046997 23071693 PMC3468442

[2] Elliott I, Pearson I, Dahal P, Thomas NV, Roberts T, Newton PN. Scrub typhus ecology: a systematic review of *Orientia* in vectors and hosts. Parasit Vectors. 2019;12:513. 10.1186/s13071-019-3751-x 31685019 PMC6829833

[3] Wei Y, Huang Y, Luo L, Xiao X, Liu L, Yang Z. Rapid increase of scrub typhus: an epidemiology and spatial-temporal cluster analysis in Guangzhou City, Southern China, 2006-2012. PLoS One. 2014;9:e101976. 10.1371/journal.pone.0101976 25006820 PMC4090214

[4] Xu G, Walker DH, Jupiter D, Melby PC, Arcari CM. A review of the global epidemiology of scrub typhus. PLoS Negl Trop Dis. 2017;11:e0006062. 10.1371/journal.pntd.0006062 29099844 PMC5687757

[5] Maude RR, Maude RJ, Ghose A, Amin MR, Islam MB, Ali M, et al. Serosurveillance of *Orientia tsutsugamushi* and *Rickettsia typhi* in Bangladesh. Am J Trop Med Hyg. 2014;91:580–3. 10.4269/ajtmh.13-0570 25092819 PMC4155564

[6] Zhang L, Zhao Z, Bi Z, Kou Z, Zhang M, Yang L, et al. Risk factors associated with severe scrub typhus in Shandong, northern China. Int J Infect Dis. 2014;29:203–7. 10.1016/j.ijid.2014.09.019 25461664

[7] Sethi S, Prasad A, Biswal M, Hallur VK, Mewara A, Gupta N, et al. Outbreak of scrub typhus in North India: a re-emerging epidemic. Trop Doct. 2014;44:156–9. 10.1177/0049475514523761 24557641

[8] Sinha P, Gupta S, Dawra R, Rijhawan P. Recent outbreak of scrub typhus in North Western part of India. Indian J Med Microbiol. 2014;32:247–50. 10.4103/0255-0857.136552 25008815

[9] Yadav D, Chopra A, Dutta AK, Kumar S, Kumar V. Scrub typhus: an uncommon cause of pyrexia without focus. J Nepal Paediatr Soc. 2013;33:234–5. 10.3126/jnps.v33i3.8172

[10] Dorji T, Wangchuk S, Lhazeen K. Clinical characteristics of scrub typhus in Gedu and Mongar (Bhutan); 2010 [cited 2018 Apr 4]. http://www.rcdc.gov.bt/web/wp-content/uploads/2012/07/Scrub-Typhus-in-Gedu-and-Mongar.pdf

[11] Dorji K, Phuentshok Y, Zangpo T, Dorjee S, Dorjee C, Jolly P, et al. Clinical and epidemiological patterns of scrub typhus, an emerging disease in Bhutan. Trop Med Infect Dis. 2019;4:56. 10.3390/tropicalmed4020056 30934849 PMC6631561

[12] le Cessie S, Nagelkerke N, Rosendaal FR, van Stralen KJ, Pomp ER, van Houwelingen HC. Combining matched and unmatched control groups in case-control studies. Am J Epidemiol. 2008;168:1204–10. 10.1093/aje/kwn236 18836151

[13] Lyu Y, Tian L, Zhang L, Dou X, Wang X, Li W, et al. A case-control study of risk factors associated with scrub typhus infection in Beijing, China. PLoS One. 2013;8:e63668. 10.1371/journal.pone.0063668 23691083 PMC3653850

[14] Pote K, Narang R, Deshmukh P. Diagnostic performance of serological tests to detect antibodies against acute scrub typhus infection in central India. Indian J Med Microbiol. 2018;36:108–12. 10.4103/ijmm.IJMM_17_405 29735837

[15] Blacksell SD, Tanganuchitcharnchai A, Nawtaisong P, Kantipong P, Laongnualpanich A, Day NP, et al. Diagnostic Accuracy of the InBios Scrub Typhus Detect Enzyme-Linked Immunoassay for the Detection of IgM Antibodies in Northern Thailand. Clin Vaccine Immunol. 2015;23:148–54. 10.1128/CVI.00553-15 26656118 PMC4744921

[16] Gupta N, Chaudhry R, Thakur CK. Determination of cutoff of ELISA and immunofluorescence assay for scrub typhus. J Glob Infect Dis. 2016;8:97–9. 10.4103/0974-777X.188584 27621559 PMC4997800

[17] Rahi M, Gupte MD, Bhargava A, Varghese GM, Arora R. DHR-ICMR Guidelines for diagnosis & management of Rickettsial diseases in India. Indian J Med Res. 2015;141:417–22. 10.4103/0971-5916.159279 26112842 PMC4510721

[18] Mørch K, Manoharan A, Chandy S, Chacko N, Alvarez-Uria G, Patil S, et al. Acute undifferentiated fever in India: a multicentre study of aetiology and diagnostic accuracy. BMC Infect Dis. 2017;17:665. 10.1186/s12879-017-2764-3 28978319 PMC5628453

[19] Pulami TM. Value chain development and technology of large cardamom and ginger in Bhutan. In: Pandey PR, Pandey IR, editors. Challenges and opportunities in value chain of spices in south Asia. Dhaka (Bangladesh): SAARC Agriculture Centre, Indian Institute of Spices Research; 2017. p. 38–55.

[20] Ogawa M, Hagiwara T, Kishimoto T, Shiga S, Yoshida Y, Furuya Y, et al. Scrub typhus in Japan: epidemiology and clinical features of cases reported in 1998. Am J Trop Med Hyg. 2002;67:162–5. 10.4269/ajtmh.2002.67.162 12389941

[21] Thomas L, Bhat A, Cheriyan H, Nirmal Babu K. Value chain development and technology practices of spices crop in India (cardamom, ginger, turmeric, black pepper and cinnamon). In: Pandey PR, Pandey IR, editors. Challenges and opportunities in value chain of spices in south Asia. Dhaka (Bangladesh): SAARC Agriculture Centre, Indian Institute of Spices Research; 2017. p. 56–115.

[22] Ansari A. Value chain development and technology practices of spice crop (cardamom (small and large), ginger, turmeric, black pepper, and cinnamon) in Nepal. In: Pandey PR, Pandey IR, editors. Challenges and opportunities in value chain of spices in south Asia. Dhaka (Bangladesh): SAARC Agriculture Centre, Indian Institute of Spices Research; 2017. p. 116–135.

[23] Mina SS, Kumar V, Chhapola V. Emerging infections in children in north India: scrub typhus. J Pediatr Infect Dis. 2017;12:114–8. 10.1055/s-0037-1599835

[24] Upadhyaya BP, Shakya G, Adhikari S, Rijal N, Acharya J, Maharjan L, et al. Scrub typhus: an emerging neglected tropical disease in Nepal. J Nepal Health Res Counc. 2016;14:122–7. 27885295

[25] Sharma PK, Ramakrishnan R, Hutin YJ, Barui AK, Manickam P, Kakkar M, et al. Scrub typhus in Darjeeling, India: opportunities for simple, practical prevention measures. Trans R Soc Trop Med Hyg. 2009;103:1153–8. 10.1016/j.trstmh.2009.02.006 19286238

[26] Vallée J, Thaojaikong T, Moore CE, Phetsouvanh R, Richards AL, Souris M, et al. Contrasting spatial distribution and risk factors for past infection with scrub typhus and murine typhus in Vientiane City, Lao PDR. PLoS Negl Trop Dis. 2010;4:e909. 10.1371/journal.pntd.0000909 21151880 PMC2998433

[27] Thiga JW, Mutai BK, Eyako WK, Ng’ang’a Z, Jiang J, Richards AL, et al. High seroprevalence of antibodies against spotted fever and scrub typhus bacteria in patients with febrile Illness, Kenya. Emerg Infect Dis. 2015;21:688–91. 10.3201/eid2104.141387 25811219 PMC4378494

[28] Faccini JL, Santos AC, Santos SB, Jacinavicius FC, Bassini-Silva R, Barros-Battesti DM. Trombiculiasis in domestic goats and humans in the state of Maranhão, Brazil. Rev Bras Parasitol Vet. 2017;26:104–9. 10.1590/s1984-29612016088 28146153

